# Comparison of Neural Recovery Effects of Botulinum Toxin Based on Administration Timing in Sciatic Nerve-Injured Rats

**DOI:** 10.3390/toxins16090387

**Published:** 2024-09-05

**Authors:** Minsu Seo, Seokjoon Hwang, Tae Heon Lee, Kiyeun Nam

**Affiliations:** Department of Physical Medicine & Rehabilitation, Dongguk University College of Medicine, Goyang 10326, Republic of Korea; seoms87@dumc.or.kr (M.S.); seokjoon.h@dumc.or.kr (S.H.); taeheon320@dumc.or.kr (T.H.L.)

**Keywords:** botulinum toxin, peripheral nerve injury, neural regeneration, functional recovery

## Abstract

This study aimed to assess the effects of the timing of administering botulinum neurotoxin A (BoNT/A) on nerve regeneration in rats. Sixty 6-week-old rats with a sciatic nerve injury were randomly divided into four groups: the immediately treated (IT) group (BoNT/A injection administered immediately post-injury), the delay-treated (DT) group (BoNT/A injection administered one week post-injury), the control group (saline administered one week post-injury), and the sham group (only skin and muscle incisions made). Nerve regeneration was assessed 3, 6, and 9 weeks post-injury using various techniques. The levels of glial fibrillary acid protein (GFAP), astroglial calcium-binding protein S100β (S100β), growth-associated protein 43 (GAP43), neurofilament 200 (NF200), and brain-derived neurotrophic factor (BDNF) in the IT and DT groups were higher. ELISA revealed the highest levels of these proteins in the IT group, followed by the DT and control groups. Toluidine blue staining revealed that the average area and myelin thickness were higher in the IT group. Electrophysiological studies revealed that the CMAP in the IT group was significantly higher than that in the control group, with the DT group exhibiting significant differences starting from week 8. The findings of the sciatic functional index analysis mirrored these results. Thus, administering BoNT/A injections immediately after a nerve injury is most effective for neural recovery. However, injections administered one week post-injury also significantly enhanced recovery. BoNT/A should be administered promptly after nerve damage; however, its administration during the non-acute phase is also beneficial.

## 1. Introduction

Peripheral nerve injuries are associated with high morbidity and a substantial socioeconomic burden, representing a significant medical challenge [1]. These injuries, caused by trauma, compression, or various pathological conditions, can lead to sensory loss, motor dysfunction, and chronic pain [2]. Peripheral nerve regeneration involves a complex sequence of events, including Wallerian degeneration, axonal regeneration, and remyelination [3]. The peripheral nervous system possesses some regenerative capacity; however, this process is often slow and incomplete, particularly in patients with a severe or chronic injury [4,5]. Thus, there is an urgent requirement to develop effective therapeutic strategies. The current strategies for the treatment of peripheral nerve injuries include surgical interventions (nerve suturing, grafting, and transfer) and conservative approaches (physical therapy, occupational therapy, and electrical stimulation) [6,7]. Surgical approaches are invasive; furthermore, they do not guarantee successful outcomes. In contrast, conservative treatment strategies often exhibit limited efficacy and are associated with slow recovery rates, particularly in chronic cases [6,7].

Botulinum toxin A (BoNT/A) has emerged as a potential option for the treatment of peripheral nerve injuries in recent years [8,9]. Several studies have demonstrated the ability of BoNT/A to promote nerve regeneration and enhance functional recovery. Various underlying mechanisms, such as elevating the neurotrophic factor levels, promoting axonal regeneration, and modulating inflammatory responses, have been identified. Franz et al. reported that botulinum toxin conditioning enhanced motor axon regeneration in mouse and human preclinical models [10]. Cobianchi et al. demonstrated that botulinum neurotoxin A enhanced functional recovery in animal models following peripheral nerve injury by promoting the regeneration of myelinated fibers [11]. Marinelli et al. revealed that botulinum neurotoxin type A alleviated neuropathic pain and promoted functional recovery in animal models following peripheral nerve injury [12]. Boyd and Gordon et al. reviewed the role of neurotrophic factors and their receptors in axonal regeneration and functional recovery following peripheral nerve injury in vitro and vivo models [13].

The benefits of administering BoNT/A in the management of acute peripheral nerve injury are becoming increasingly clear; however, its potential effects on chronically damaged nerves are underexplored. Acute nerve injuries involve immediate axonal transection and Wallerian degeneration; in contrast, chronic injuries are characterized by the presence of persistent inflammatory responses, atrophy of neuronal cell bodies, and diminished regenerative capacity [14,15,16]. These distinct pathophysiological mechanisms indicate that identical therapeutic approaches may yield different outcomes in acute and non-acute scenarios. Nevertheless, it is necessary to investigate the potential beneficial effects of administering BoNT/A on chronic nerve injuries, given its demonstrated ability to promote nerve regeneration and modulate inflammatory responses. Datta et al. concluded that BoNT/A can be used to effectively manage chronic neuropathic pain [17]. Han et al. [18] also reported that BoNT/A can result in a significant alleviation of pain in patients with spinal cord injuries. A recent review suggested that BoNT/A has promising outcomes in terms of reducing pain in patients with chronic neuropathic pain [19]. Thus, it can be hypothesized that BoNT/A presents a new possibility for treating long-term neuropathies, given its ability to promote regeneration and improve function even in non-acute nerve injuries. Therefore, this study aimed to compare the nerve recovery effects observed following the administration of BoNT/A in a rat model of sciatic nerve injury. Enzyme-linked immunosorbent assays (ELISAs), immunofluorescence staining, and toluidine blue staining were performed in this study. In addition, the sciatic functional index (SFI) was assessed, and an electrophysiological test was conducted to evaluate functional recovery.

## 2. Results

### 2.1. Effect of BoNT/A on Schwann Cell Activity in Injured Sciatic Nerves Based on the Timing of BoNT/A Administration (GFAP and S100β)

**Figure 1:**
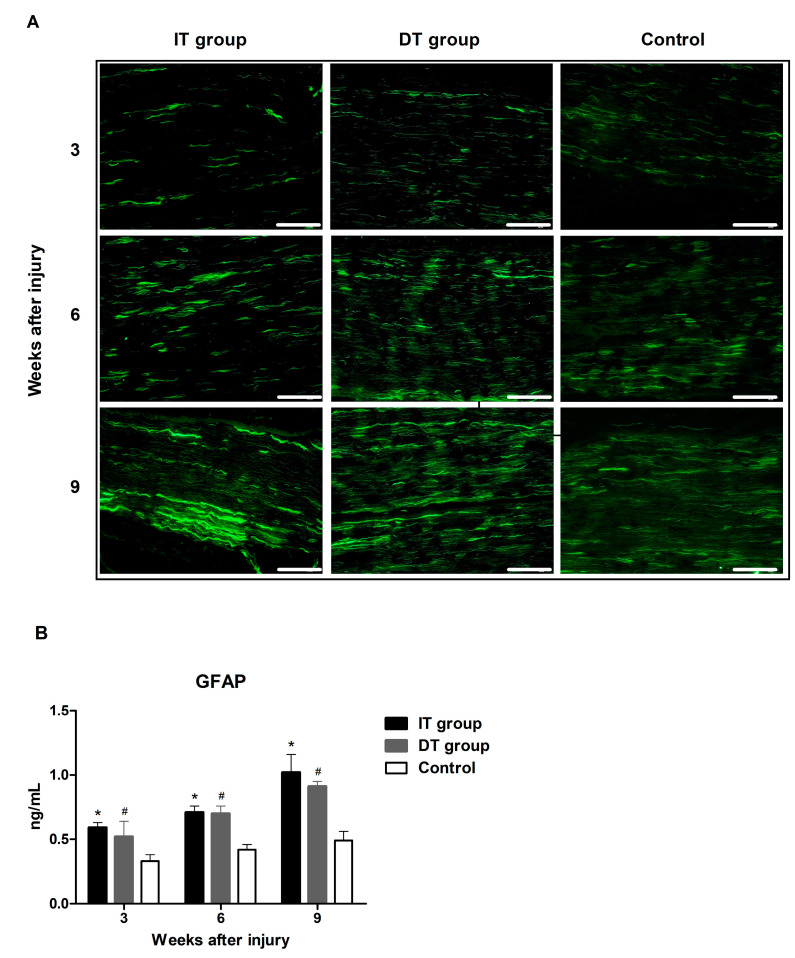
Effect of botulinum neurotoxin A (BoNT/A) on glial fibrillary acid protein (GFAP) expression. (**A**) Image depicting immunofluorescence staining (scale bar = 150 µm; 200× magnification). (**B**) The ELISA results indicate that GFAP expression in the IT and DT groups was significantly higher than that in the control at all time points. However, no differences were observed between the IT and DT groups. * indicates IT group vs. control group, # indicates DT group vs. control group (*p* < 0.05). n = 5 in each group. The error bars indicate the standard error of the mean.

**Figure 2:**
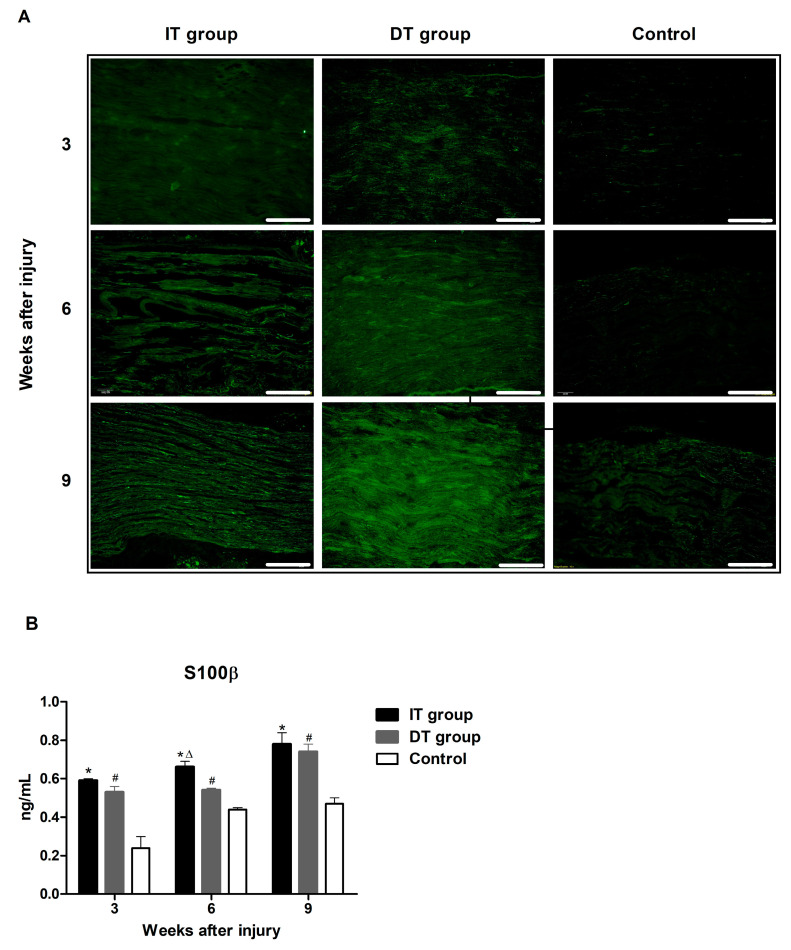
Effect of BoNT/A on astroglial calcium-binding protein S100β (S100β) expression: (**A**) Image depicting immunofluorescence staining (scale bar = 150 µm; 200× magnification). (**B**) ELISA results revealed significantly higher S100β levels in the IT and DT groups compared with that in the control, with a significant difference being observed between the IT and DT groups at week 6. * indicates IT group vs. control group, ∆ indicates IT group vs. DT group, and # indicates DT group vs. control group (*p* < 0.05). n = 5 in each group. The error bars indicate the standard error of the mean.

Immunofluorescence staining revealed increased glial fibrillary acid protein (GFAP) and astroglial calcium-binding protein S100β (S100β) expression in the IT and the DT groups (Figure 1A and Figure 2A). An ELISA revealed that the GFAP expression in the IT and DT groups at weeks 3, 6, and 9 was markedly higher than that in the control group (Figure 1B). GFAP expression in the IT group was higher than that in the DT group; however, the difference was not statistically significant. An ELISA revealed that the S100β expression in the IT and DT groups at weeks 3, 6, and 9 (Figure 2B) was significantly higher than that in the control group. The expression levels in the IT group were higher than those in the DT group; however, the difference was statistically significant only at week 6.

### 2.2. Effect of BoNT/A on Axon Regeneration in Injured Sciatic Nerves Based on the Timing of BoNT/A Administration (GAP43 and NF200)

**Figure 3:**
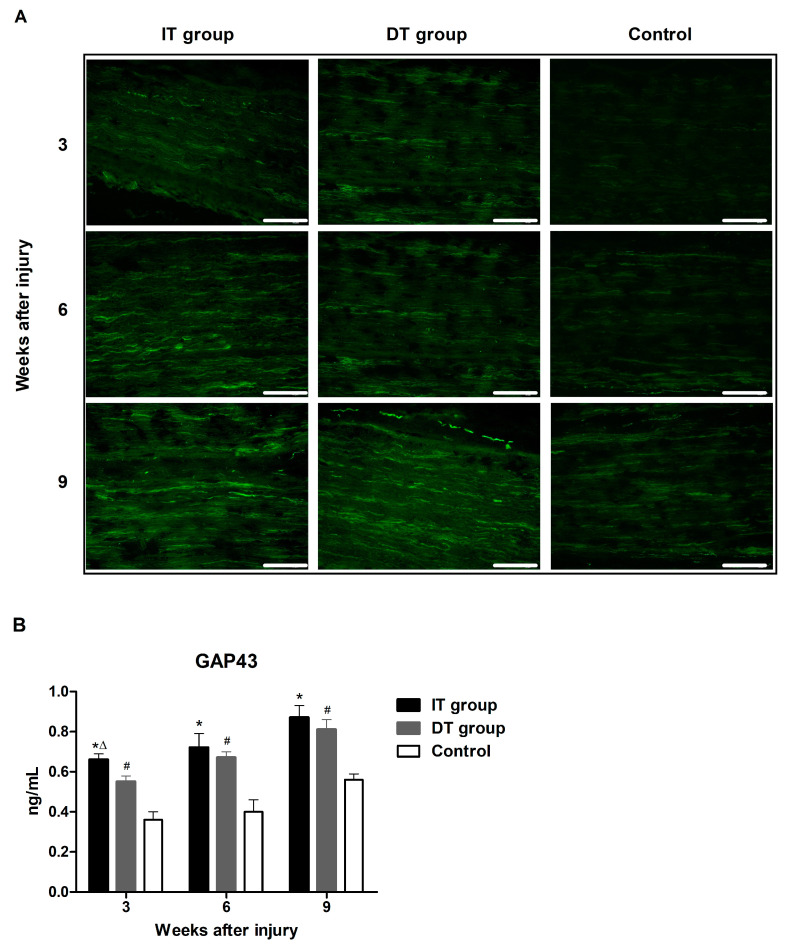
Effect of BoNT/A on growth associated protein 43 (GAP43) expression. (**A**) Image depicting immunofluorescence staining (scale bar = 150 µm; 200× magnification). (**B**) The ELISA results indicate significantly higher expression of GAP43 in the IT and DT groups compared with that in the control group at all time points, with a significant difference being observed between the IT and DT groups at week 3. * indicates IT group vs. control group, ∆ indicates IT group vs. DT group, and # indicates DT group vs. control group (*p* < 0.05). n = 5 in each group. The error bars indicate the standard error of the mean.

**Figure 4:**
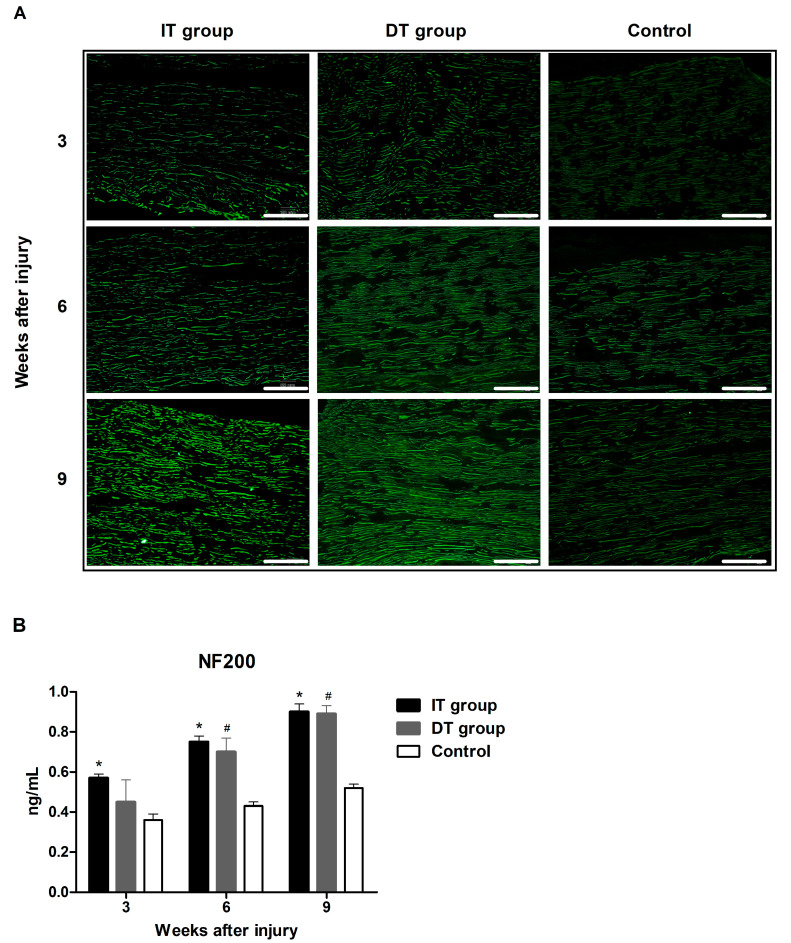
Effect of BoNT/A on neurofilament 200 (NF200) expression. (**A**) Image depicting immunofluorescence staining (scale bar = 150 µm; 200× magnification). (**B**) The ELISA results revealed that the expression of NF200 in the IT and DT groups at weeks 6 and 9 was significantly higher than that in the control group. No statistically significant difference was observed between the IT and DT groups at any time point. * indicates IT group vs. control group, # indicates DT group vs. control group (*p* < 0.05). n = 5 in each group. The error bars indicate the standard error of the mean.

Immunofluorescence staining revealed increased expression of neurofilament 200 (NF200) and growth-associated protein 43 (GAP43) in the IT and DT groups (Figure 3A and Figure 4A). An ELISA revealed that the GAP43 expression in the IT and DT groups at weeks 3, 6, and 9 was significantly higher than that in the control group (Figure 3B). The GAP43 expression in the IT group at weeks 3 and 9 was significantly higher than that in the DT group. An ELISA revealed that the expression of NF200 (Figure 4B) in the IT group at weeks 3, 6, and 9 was significantly higher than that in the control group. The expression of NF200 in the DT group at weeks 6 and 9 was significantly higher than that in the control group. The NF200 expression in the IT group was higher than that in the DT group; however, the difference was not statistically significant.

### 2.3. Effect of BoNT/A on Nerve Growth Factors in Injured Sciatic Nerves Based on the Timing of BoNT/A Administration (BDNF)

**Figure 5:**
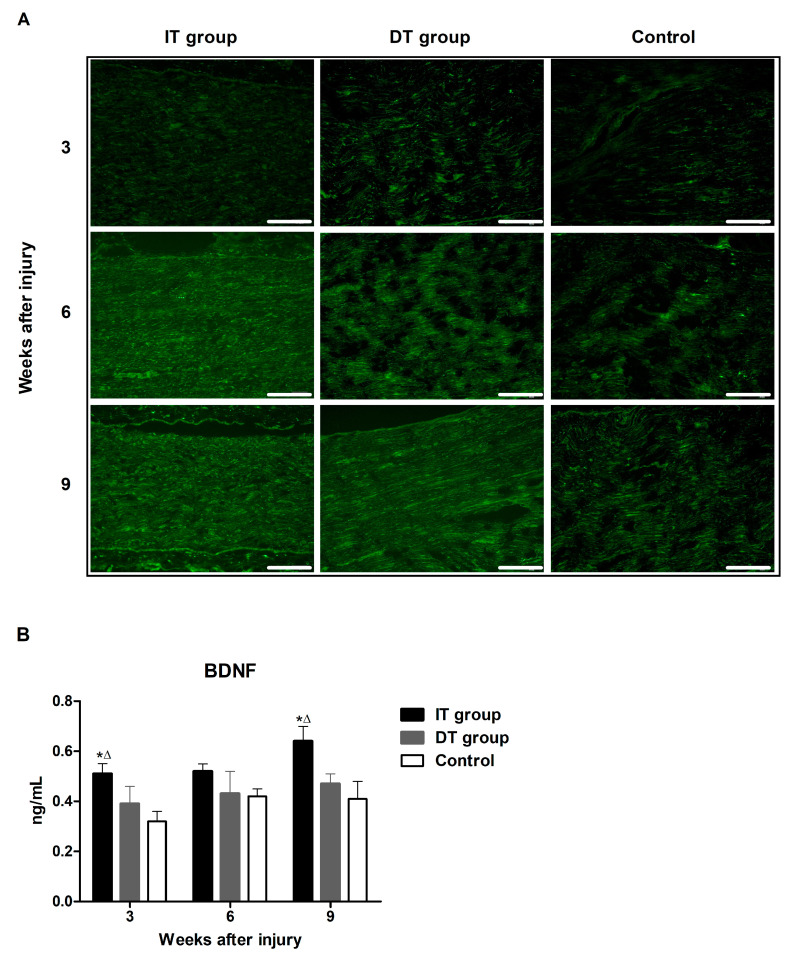
Effect of BoNT/A on brain-derived neurotrophic factor (BDNF) expression. (**A**) Image of immunofluorescence staining (scale bar = 150 µm; 200× magnification). (**B**) ELISA results indicate significantly higher BDNF levels in the IT group compared to the DT and control groups at weeks 3 and 9, with no significant differences observed between the DT group and the control group. * indicates IT group vs. control group, ∆ indicates IT group vs. DT group. n = 5 in each group. The error bars indicate the standard error of the mean.

Immunofluorescence staining revealed higher expression of brain-derived neurotrophic factor (BDNF) in the IT and the DT groups (Figure 5A). An ELISA revealed that the expression of NF200 (Figure 5B) in the IT group at weeks 3, 6, and 9 was markedly higher than that in the control group. The expression of BDNF in the IT group at weeks 3, 6, and 9 was significantly higher than that in the DT and the control groups. No significant differences were observed between the DT and control groups.

### 2.4. Effect of BoNT/A on Axonal Regeneration and Remyelination

**Figure 6:**
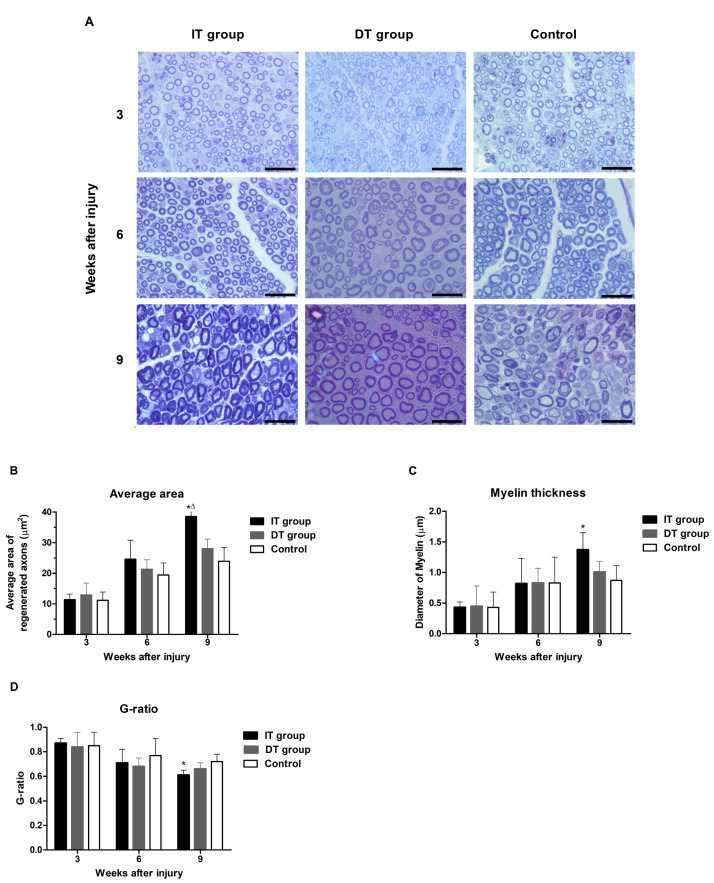
Effect of BoNT/A on axonal regeneration and remyelination. (**A**) Images depicting embedded sciatic nerve sections stained with toluidine blue (scale bar = 20 µm; 1000× magnification). (**B**) Average area, (**C**) mean diameter of the myelin sheath, and (**D**) remyelination degree of axons (G-ratio). The quantitative analysis revealed that the average axon area and myelin thickness in the IT group were significantly greater at week 9 compared with those in the DT and control groups. The G-ratio analysis revealed a significantly lower value in the IT group at week 9, indicating better remyelination. * indicates IT group vs. control group, ∆ indicates IT group vs. DT group. n = 5 in each group. The error bars indicate the standard error of the mean.

The images of toluidine blue-stained slices of implanted sciatic nerves were divided into three categories and analyzed (Figure 6A). The average area of the regenerated axons in the IT, DT, and control groups was measured at 3, 6, and 9 weeks post-injury (Figure 6B). The average area of the regenerated axon in the IT group was significantly greater than those of the DT and control groups at week 9. No statistically significant differences were observed among the three groups in terms of the average area of the regenerated axons at week 3.

A trend towards a greater average axon area compared with those in the DT and control groups was observed in the IT group by week 6; however, this difference did not reach statistical significance. The average myelin thickness of the regenerated axons was also assessed in the IT, DT, and control groups at 3, 6, and 9 weeks post-injury (Figure 6C). The myelin thickness in the IT group at week 9 was significantly greater than that of the control group. Some improvement was observed in the DT group; however, these differences did not achieve statistical significance when compared with the control group. No statistically significant differences were observed among the three groups in terms of the myelin thickness at week 3. A trend towards a greater myelin thickness compared with those in the DT and control groups was observed in the IT group by week 6; however, this difference did not reach statistical significance. The degree of remyelination of the regenerated axons (G-ratio) was assessed in the IT, DT, and control groups at 3, 6, and 9 weeks post-injury (Figure 6D). The G-ratio in the IT group at week 9 was significantly lower than that in the control group. The DT group exhibited a trend toward improved myelination; however, this difference did not achieve statistical significance when compared with the control group. No statistically significant differences were observed among the three groups in terms of the G-ratio at weeks 3 and 6.

### 2.5. Effects of BoNT/A on Functional Recovery Based on the Timing of BoNT/A Administration

#### 2.5.1. Compound Action Potential Amplitude 

**Figure 7:**
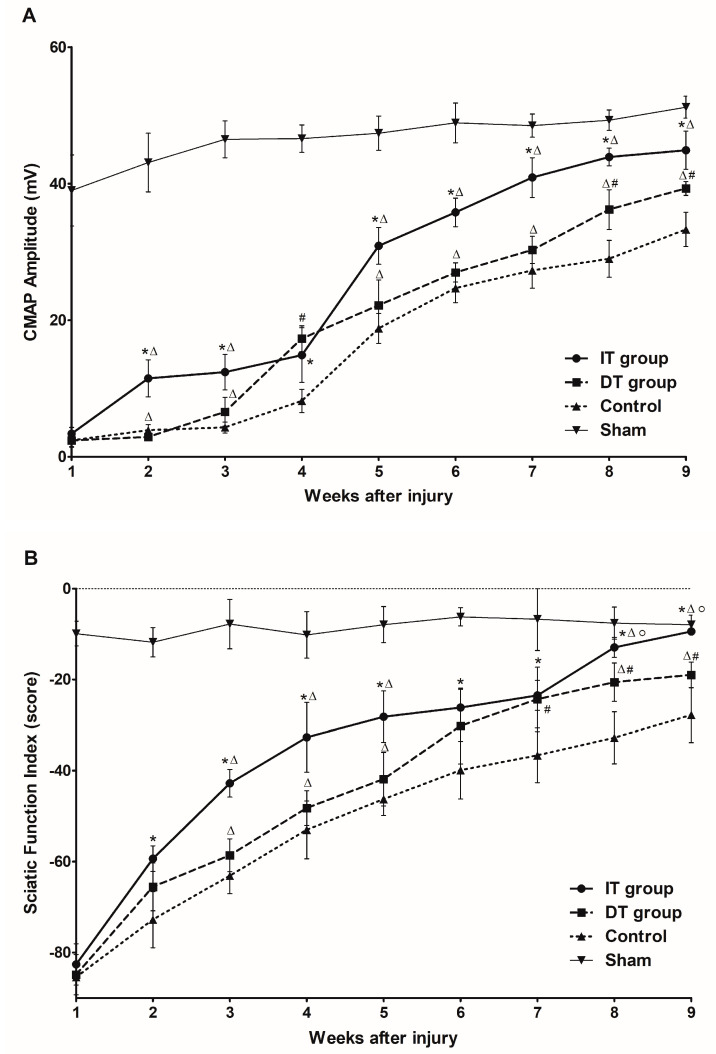
Effect of BoNT/A on functional recovery. (**A**) CMAP amplitude (mV) and (**B**) SFI (score) were measured weekly for up to 9 weeks following neural damage (n = 5 per group). (**A**) The CMAP amplitude in the IT group was significantly greater than that in the control group starting from week 2 after injury (*p* < 0.05). No statistically significant differences were observed between the DT and control groups until week 7; however, significantly greater CMAP values were observed from week 8 onwards (*p* < 0.05). (**B**) The SFI scores exhibited similar trends, with the IT group exhibiting significantly improved functional recovery compared with the control group starting from week 2 and the DT group exhibited significant improvements starting from week 7. The IT group exhibited results comparable with those of the sham group by week 9. * indicates IT group vs. control group, ◦ indicates IT group vs. sham group, ∆ indicates IT group vs. DT group, and # indicates DT group vs. control group (*p* < 0.05). n = 5 in each group. The error bars indicate the standard error of the mean.

The electrophysiological study revealed that the compound muscle action potential (CMAP) amplitude in the IT group was significantly greater than that in the control group two weeks after the nerve-crushing injury (Figure 7A). No significant differences were observed between the DT and control groups until week 7; however, a significant difference in CMAP amplitude was observed from week 8 onwards. A comparison between the IT and DT groups revealed that the CMAP amplitude in the IT group was significantly greater than that in the DT group at every time point, except for week 4. The CMAP values of all the groups were lower than that of the sham group at week 9.

#### 2.5.2. Sciatic Function Index

The SFI analysis results exhibited trends similar to those of the CMAP results. The SFI score in the IT group was significantly higher than that in the control group starting from week 2 (Figure 7B). The SFI score in the DT group was significantly higher than that in the control group at week 7 (*p* < 0.05). A comparison between the IT and DT groups revealed that the SFI score in the IT group was significantly higher throughout the experiment, with statistically significant differences being observed at weeks 3–5 and after week 8. The SFI results exhibited no statistically significant distinction between the IT group and the sham group after week 8.

## 3. Discussion

This study compared the neural recovery effects of the timing of administration of BoNT/A in a rat sciatic nerve injury model. The immediate treatment (IT) group, which received BoNT/A immediately after the nerve injury, exhibited superior results in terms of nerve regeneration and functional recovery. Significant recovery effects were also observed in the delayed treatment (DT) group, which received BoNT/A one week post-injury. These results suggest that the administration of BoNT/A can promote nerve regeneration during the acute phases of nerve injury, thereby providing important insights into the timing and effectiveness of administering BoNT/A in the management of peripheral nerve injury. In particular, these findings indicate the potential for treatment during non-acute stages. However, it must be noted that these findings were derived from experiments conducted on rats, not humans.

Schwann cells, which play a crucial role in the nerve regeneration process, are key players that facilitate the recovery of damaged nerves [20]. The administration of BoNT/A can stimulate the proliferation of Schwann cells, thereby promoting the process of peripheral regeneration following nerve injury [21]. GFAP and S100β are markers of myelinating and non-myelinating Schwann cells, respectively [22,23,24]. The expression of these markers was increased in the present study, indicating that the administration of BoNT/A promotes Schwann cell activation. This effect was more pronounced in the IT group, suggesting that early intervention may enhance the response of Schwann cells. However, a significant increase was also observed in the DT group compared with that in the control group. The expression of GAP43 (which plays an important role in nerve growth and regeneration) and NF200 (an indicator of the structural stability of mature nerve fibers) [11] were elevated in the IT and DT groups, indicating an increase in axonal regeneration. Consistently higher GAP43 and NF200 expression levels were observed in the IT group at early and late time points, suggesting that the early administration of BoNT/A can promote stronger and more sustained axonal growth. These results are consistent with those reported by Franz et al. and Cobianchi et al., who proposed that the administration of BoNT/A enhances motor axon regeneration and promotes myelinated fiber regeneration [10,11]. A significant increase was also observed in the DT group compared with the control group. BDNF plays a vital role in neuronal survival, growth, and maintenance, thereby promoting the regeneration of damaged nerves [13]. The increase in BDNF expression in the IT group indicates that the administration of BoNT/A stimulates the production of neurotrophin, which is more effective when administered early. This supports the findings of the review by Boyd and Gordon, which evaluated the roles of neurotrophin and highlighted its critical roles in functional recovery and axonal regeneration [13]. A significant increase was also observed in the DT group compared with the control group; however, the difference was not statistically significant. Toluidine blue staining revealed more pronounced improvements in the axon area, myelin thickness, and G-ratio in the IT group, demonstrating that the early administration of BoNT/A promotes structural nerve regeneration. The significant improvement observed in the IT group at week 9 suggests that the administration of BoNT/A has long-term effects. Improvements in the axon area, myelin thickness, and G-ratio were also observed in the DT group; however, these did not reach statistical significance. The CMAP and SFI results revealed that the fastest and most effective recovery occurred in the IT group. However, significant improvements were also observed in the DT group compared with the control group. Significant differences were observed between the DT and control groups 7–8 weeks after treatment. This finding suggests that the delayed administration of BoNT/A can also aid in long-term functional recovery. 

Wallerian degeneration commences at the injury site immediately after nerve injury [23]. Chen et al. reported that damaged axons degrade during this process. In addition, Schwann cells realign to support the regrowth of new axons [25]. Effective suppression of the inflammatory response during the early stages of Wallerian degeneration was observed in the IT group, where BoNT/A was administered immediately after nerve injury [11]. This may have reduced the extent of edema and secondary damage at the injury site, thereby promoting Schwann cell realignment and optimizing axon regeneration [21,26,27,28]. Marinelli et al. [12] and Cobianchi et al. [11] suggested that the regenerative effects of BoNT/A after nerve injury can be attributed to the ability of the neurotoxin to enhance the normal response of Schwann cells to injury. These mechanisms may have acted synergistically, leading to the faster and more efficient functional recovery observed in the IT group. Significant recovery effects were also observed in the DT group, which received BoNT/A one week after the injury. This finding suggests that the administration of BoNT/A can promote nerve regeneration even in the non-acute phase. Inflammation persists during the non-acute phase; however, it is less severe than that observed during the early stage. BoNT/A may further suppress any remaining inflammation, maintain Schwann cell activity, and promote the expression of neurotrophic factors and axon growth-related proteins at this point. These mechanisms may have led to the significant functional recovery observed in the DT group.

Most studies using BoNT/A for nerve regeneration following central and peripheral nervous system injuries focused on the acute phase. Vacca et al. [29] reported that the early spinal administration of BoNT/A induced long-lasting neuroprotection, promoted motor function recovery, and alleviated neuropathic pain in a murine model of severe spinal cord injury. Several studies have evaluated the effects of administering BoNT/A immediately after peripheral nerve injury or at the onset of neuropathic pain [9]. The outcomes observed in the DT group in the present study hold significant clinical implications as they demonstrate the efficacy of administering BoNT/A even one week post-nerve injury. Immediate treatment is typically possible in clinical settings following nerve injury; however, the commencement of specialized treatments such as the administration of BoNT/A may face practical delays. These delays can arise from the time required for the accurate diagnosis of nerve damage, specialist consultations, patient consent processes, drug preparation and procurement, or the prioritization of treatments for concomitant life-threatening injuries. The findings of the present study suggest that the administration of BoNT/A may be beneficial even in cases wherein nerve injury was sustained previously. These observations present a new avenue for the treatment of non-acute nerve injuries, which currently rely primarily on surgical interventions and conservative approaches. The expansion of the application of BoNT/A treatment may pave the way for more diverse and effective treatment options for patients with nerve injuries. Furthermore, these findings may aid clinicians in formulating and implementing treatment plans with greater flexibility.

This study has several limitations. First, the sample size posed significant constraints. Sixty Sprague Dawley (SD) rats were used in the present study; however, the number of animals per experimental group remained relatively small. This may have potentially limited the statistical power and prevented some results from achieving statistical significance. Second, the rate of nerve regeneration in rats is higher than that in humans. This may have limited the direct application of the results of the rat model to humans. For instance, the rate of nerve regeneration observed one week after nerve injury in a rat may correspond to that observed several weeks or months later in humans [30,31]. Thus, the effects of BoNT/A administered at the same time point could differ between species. The present study compared the effects of BoNT/A administered immediately after nerve injury and one week later; however, these results are specific to rats. Further studies must be conducted in the future to explore the effects of BoNT/A during the acute, subacute, and chronic phases of nerve injury and apply these findings to humans. The best timing for commencing BoNT/A treatment must be determined clinically by evaluating its effects at different stages and developing appropriate treatment protocols. Third, it may not be appropriate to directly apply the dosage results from animal models to humans, considering the physiological differences between the rat model used in the present study and humans. The higher doses of BoNT/A used in animal experiments account for the differences in body mass, metabolic rate, and pharmacological responses. These factors must be considered carefully when adjusting the dose for human use. Doses for the treatment of spasticity typically range from 1 to 12 U/kg in clinical practice, corresponding to a maximum total dose of 15 IU/kg or up to 400 units per injection site in adults [32]. Thus, additional studies must be conducted to determine the appropriate dose for humans based on the foundational data obtained from this study. Lastly, there are limitations in the protocol for the administration of BoNT/A used in the present study. The present study focused solely on a single dose of BoNT/A, neglecting to explore the effects of other dosing regimens. Further studies must be conducted to determine the optimal injection frequency and intervals between repeated injections to develop a comprehensive protocol for the administration of botulinum toxin following a nerve injury.

## 4. Conclusions

This study compared the effects of neural recovery in rats with a sciatic nerve injury based on the timing of botulinum toxin administration. The findings of the present study indicate that the administration of BoNT/A injections immediately after a nerve injury was the most effective. Injections administered a week after injury resulted in a faster recovery compared with the control group. Thus, BoNT/A injections should be administered at the earliest time point to facilitate effective recovery after nerve damage. Furthermore, BoNT/A administered during the non-acute phase may also be effective, given that it was effective even when injected a week later. The findings of this study provide an important foundation for evaluating the potential of BoNT/A as a therapeutic agent in nerve regeneration.

## 5. Materials and Methods

### 5.1. Animals and Surgical Procedure

Sixty SPF SD rats were divided into four groups and housed in standard cages upon arrival. A 12 h light/dark cycle was maintained from 6:30 to 18:00. The temperature was maintained at 23 ± 1 °C. The rats weighed 189–211 g and were approximately 6 weeks old at the time of the procedure. The animal research was conducted in accordance with the guidelines provided in the ‘Guide for the Care and Use of Laboratory Animals’ (Institute for Laboratory Animal Research, National Research Council, The National Academies, Washington, DC, USA, 2011). The animal care and experimental procedures were reviewed and approved by the Institutional Animal Care and Use Committee at Dongguk University (protocol code 202306250; approval date: 6 July 2023).

Anesthesia was induced using 1–2% isoflurane. A mid-thigh incision was made to expose the right sciatic nerve, which was exposed after a blunt dissection of the muscle using fine surgical scissors and forceps and an incision through the skin beneath the hip. The sciatic nerve was compressed 1 cm proximal to its bifurcation into the tibial, common peroneal, and sural branches. A Halsey needle holder (AE 064/13, NOPA, Tuttlingen, Germany) was used to crush the sciatic nerve for 30 s [33]. The rats were returned to their heated cages after VICRYL 3-0 (W9114, Ethicon LLC, Bridgewater Township, NJ, USA) wound closure until their reflexes recovered.

Onabotulinum toxin A (BOTOX^®^, Allergan Inc., Irvine, CA, USA) was injected into the nerve injury site using a 50 μL Hamilton syringe (HAMILTON CO., NO706, Hamilton, OH, USA). BoNT/A was injected at a dose of 7 U/kg, which was determined to be the most appropriate dose for neural regeneration based on the findings of previous studies [34]. A stock solution of BoNT/A (100 U/mL), where 100 units of botulinum toxin are equivalent to approximately 5 ng of toxin [35], was freshly prepared by diluting it in 0.5 mL of normal saline (0.9% NaCl). A single intraneural BoNT/A injection was performed as scheduled.

### 5.2. Experimental Groups

**Figure 8:**
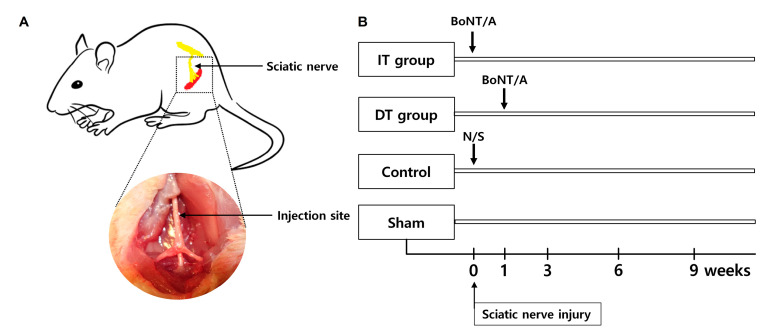
(**A**) Schematic diagram. (**B**) Experimental protocols.

The rats with crushed sciatic nerves were divided into four groups: the immediately treated (IT), delay-treated (DT), control, and sham groups. A single intraneural injection of BoNT/A (7 U/kg) was administered immediately after the nerve-crushing injury in the IT group. The injured area was reopened one week after the nerve injury in the DT group, and the same BoNT/A injection was administered before suturing the wound. An intraneural injection of normal saline was administered one week after the nerve-crushing injury in the control group. Only skin and muscle incisions were made in the sham group. Sciatic nerves were harvested from all groups, with the exception of the sham group, at weeks 3, 6, and 9. All intraneural injections were administered by an expert to avoid the possibility of mechanical nerve injury (Figure 8).

### 5.3. Neurophysiology Test

The nerve function and muscle response after a nerve crush injury were evaluated over a period of nine weeks by conducting serial electrophysiological investigations. The CMAP values were recorded from animals anesthetized with 1–2% isoflurane. Percutaneous stimulation of the sciatic nerve was conducted by delivering single 0.3 ms pulses using a pair of bar electrodes placed at the sciatic notch. The CMAP of the gastrocnemius medialis muscle was recorded using microneedle electrodes. The reference electrode was placed at the site of insertion of the Achilles tendon. The ground electrode was placed on the tail. All potentials were visualized using electromyography equipment (Synergy, version 11.0; Viasys Healthcare, Conshohocken, USA) to assess the amplitude. The stimulus intensity was raised slowly until supra-maximal stimulation was achieved to record the CMAP amplitudes and ensure that the maximum response was captured. This methodology facilitated consistent and reproducible assessments of neural and muscular recovery throughout the study period [36].

### 5.4. Behavioral Tests 

Individual gait patterns were analyzed to evaluate the progressive recovery of motor function. The SFI score was calculated using multiple footstep parameters. The rats were made to roam in a 12 × 12 × 60 cm Perspex runway corridor with their hind paws inked black. The footprints were recorded. A minimum of five footsteps were captured on each of the three tracks to calculate the footstep characteristics. The rats were observed once a week from the first to the ninth week. The following parameters were assessed in the footstep analysis: print length (PL), defined as the distance between the heel and the third toe; toe spread (TS), defined as the distance between the first and fifth toes; and intermediate toe spread (ITS), defined as the distance between the second and fourth toes. Experimental (E) and normal (N) data were collected for the right and left footsteps. The SFI score was computed using the subsequent formula: [SFI = −38.3 × (EPL-NPL)/NPL + 109.5 × (ETS-NTS)/NTS + 13.3 × (EITS-NITS)/NITS − 8.8] [37]. The values of −38.3 for PL, 109.5 for TS, and 13.3 for ITS used in this formula are coefficients that reflect the relative importance of each parameter in assessing nerve damage. The value of −8.8 is a constant used for baseline adjustment in the SFI calculation. The SFI score of a normal rat is 0, whereas that of a severely injured rat approaches −100.

### 5.5. Immunostaining of the Sciatic Nerve

The rats were administered 1–2% isoflurane (Sigma-Aldrich, St. Louis, MO, USA) at weeks 3, 6, and 9. A 10 mm sciatic nerve segment distal to the injury site was excised, embedded in OCT compound, and frozen in liquid nitrogen. The frozen tissue block was stored at −80 °C and sectioned into 8 μm slices for immunofluorescence staining. These slices were fixed with 4% paraformaldehyde (Sigma-Aldrich, Milan, Italy), permeabilized with 0.5% Triton X-100, and blocked with 5% bovine serum albumin (Sigma-Aldrich, Milan, Italy). Primary antibodies from Cell Signaling Technology, Inc. (Danvers, MA, USA) were applied and the sections were incubated overnight at 4 °C. The expression of glial fibrillary acid protein (GFAP) and astroglial calcium-binding protein S100β (S100β), two proteins that are expressed by non-myelinating and myelinating Schwann cells, respectively, enabled the evaluation of the effect of BoNT/A on the activity of the Schwann cells of a damaged sciatic nerve [38]. The expression of growth-associated protein 43 (GAP43), and neurofilament 200 (NF200) were measured to assess axonal regrowth; in addition, the expression of brain-derived neurotrophic factor (BDNF) was also measured [11,39,40,41]. The primary antibodies applied in the experiment included GFAP (diluted 1:500), S100β (diluted 1:200), GAP43 (diluted 1:250), NF200 (diluted 1:500), and BDNF (diluted 1:250). The slides were washed with PBS and treated with diluted secondary antibodies (anti-mouse IgG and anti-rabbit IgG2 at a dilution of 1:500 to 1:1000). The slides were stored at room temperature in a humid room for an hour on the next day. The samples were rinsed again in PBS, mounted with coverslips, and examined using a confocal microscope (STELLARIS, Leica Microsystems, Wetzlar, Germany).

### 5.6. ELISA

ELISA kits for GFAP [CUSABIO, CSB-E08602r], S100β [CUSABIO, CSB-E08066r], GAP43 [CUSABIO, CSB-EL009231RA], NF200 [MyBioSource, MBS3808264], and BDNF [CUSABIO, CSB-E04685r] were used in accordance with the manufacturer’s instructions to measure the levels of these proteins in the frozen sciatic nerve tissue. The expression of nerve regeneration proteins was analyzed.

### 5.7. Toluidine Blue Staining

A solution containing 0.1 M Sorensen’s phosphate buffer (pH 7.2), 4% paraformaldehyde (15754-S, EMS), and 2% glutaraldehyde (16216, EMS) was used to fix 5 mm nerve segments for 48 h at 4 °C. The samples were dehydrated in alcohol, starting at a concentration of 50%, and embedded in Polyscience’s Araldite 1502 resin, and then fixed with 2% osmium tetroxide. The tissues were sectioned into 1 μm slices using an Ultracut E microtome (Reichert Inc., Buffalo, NY, USA) and stained with 1% toluidine blue for enhanced contrast. Five random sample locations per section, spaced 2 mm apart, were analyzed using an Eclipse Ci-L microscope (Nikon, Tokyo, Japan) at 1000× magnification. The average area of the regenerated nerves, the mean diameter of the myelin sheath, and the axon-to-fiber diameter ratio (G-ratio) were measured to assess axonal regeneration. These parameters facilitated a comprehensive assessment of neural recovery and remyelination efficiency. Non-overlapping micrographs loaded into ImageJ software (FIJI Package, version 2.0, NIH, Bethesda, MD, USA) were used to perform a thorough quantitative analysis. The measurements included axons contacting the margins to guarantee precision and completeness. An automatic thresholding process was used to generate a binary image, allowing for clear distinctions between axonal and myelin structures. Lines were drawn between the fibers where the myelin sheaths touched to segment them individually, thereby ensuring the precise measurement of each fiber. Statistical analysis was carried out with a threshold of 2 μm^2^ for the minimum particle size and a circularity of 0.2, ensuring that only significant particles were included and minor debris was excluded. This stringent filtering process ensured that only relevant neural structures were included in the final data, thereby providing a reliable measure of axonal regeneration and myelination status. The processed samples were analyzed to determine the extent of neural recovery, thereby providing valuable data regarding the overall assessment of the experimental treatments.

### 5.8. Statistical Analysis

The results are expressed as mean values with either the standard deviation or standard error of the mean. Differences among the groups at each time point were analyzed using one-way ANOVA followed by Tukey’s multiple comparison test, using GraphPad Prism version 9.5.1 for Windows (GraphPad Software, Boston, MA, USA). Statistical significance was set at *p* < 0.05.

## Data Availability

Data are contained within the article.

## Abbreviations

BoNT/A	Botulinum neurotoxin A
BDNF	Brain-derived neurotrophic factor
CMAP	Compound muscle action potential
ELISA	Enzyme-linked immunosorbent assay
GAP43	Growth-associated Protein 43
GFAP	Glial fibrillary acid protein
NF200	Neurofilament 200
PBS	Phosphate-buffered saline
S100β	Astroglial calcium-binding protein S100β
SFI	Sciatic functional index
